# Criteria for assessing the quality of clinical practice guidelines in paediatrics and neonatology: a mixed-method study

**DOI:** 10.1186/s12911-021-01628-1

**Published:** 2021-09-21

**Authors:** Joanna Dagard, Nadia Mazille-Orfanos, Nawras Georgi, Intissar Dechicha, Guy Carrault, Patrick Pladys, Alain Beuchée

**Affiliations:** 1 Department of Pediatrics/Neonatology, CHU Rennes, 35033 Rennes, France; 2 LTSI-UMR_S 1099, Univ Rennes, Inserm, 35000 Rennes, France; 3 Paediatric Department, Hospital of Fougères, Fougères, France

**Keywords:** Quality criteria, Neonatology, Paediatrics, Guidelines, Professional practice, Clinical practice guidelines” (CPG), Qualitative study, Mixed-methods

## Abstract

**Background:**

Evidenced-based practice is a key component of quality care. This study aims to explore users’ expectations concerning paediatric local clinical practice guidelines.

**Methods:**

A mixed method approach was applied, including material from quantitative questionnaire and semi-structured interviews. Data were analysed using descriptive statistics and qualitative content analysis. Data were analysed with constant comparative method. Qualitative data were parsed and categorized to identify themes related to decision-making.

**Results:**

A total of 83 physicians answered the survey (response rate 83%). 98% of the participants wanted protocols based on international guidelines, 80% expected a therapeutic content. 24 semi-structured interviews were conducted to understand implementation processes, barriers and facilitators. Qualitative analysis revealed 5 emerging themes: improvement of local clinical practice guidelines, patterns of usage, reasons for non-implementation, alternative sources and perspectives.

**Conclusion:**

Some criteria should be considered for the redaction of local clinical practice guidelines: focus on therapeutic, ease of access, establish local clinical practice guidelines based on international guidelines adapted to the local setting, document references and include trainees such as residents in the redaction.

**Supplementary Information:**

The online version contains supplementary material available at 10.1186/s12911-021-01628-1.

## Background

Evidence-based medicine and practice are increasingly required by professional associations [1]. In addition, introduction of quality approach in the hospital is now part of national strategies for healthcare. It aims at standardisation of practices to reduce the risk of error and the cost of care [2]. Several hospitals in the world already have ISO 9001:2000 certifications which consist in generic standards that define requirements and guidelines for quality management systems [3]. In this current context, more and more clinical guidelines are being developed [1].

Clinical practice guidelines (CPGs) are intended to improve patient management and care [4] with the primary goal of improving patient health [5]. CPGs help to ensure that patients and physicians are provided with current and robust empirical evidence to adequately inform their decisions and guide their clinical practices.

However, these guidelines need to be adjusted locally according to local preferences and resources, while maintaining high-quality evidence-based recommendations [6].

The ADAPTE Working Group (mainly oncologists) published in 2006 a literature review and proposed a protocol to implement and adapt clinical guidelines locally. The objective was the transposition of guidelines from one cultural and organizational setting to another (the authors call it “trans-contextual adaptation”). This high-quality protocol was developed to decrease workload and inefficient use of resources when adapting guidelines, as opposed to a guideline made from scratch [7].

In spite of all these efforts, recommendations described in guidelines are not necessarily followed [1], for example Grol et al. found in an observational study that only 61% of general practitioners followed guidelines for relevant decisions [8].

Many authors have questioned the factors involved in the implementation of guidelines, such as Francke et al. in a meta-analysis of twelve systematic reviews [1]. However, few studies have considered these issues in the case of local CPGs. The originality of our study is the use of a mixed methodology including a quantitative approach combined with a qualitative method, and also the fact that we directly questioned the point of view of the guidelines users.

The main objective of this study is to explore what physicians expect from local CPGs in order to identify a set of criteria to assist in the development of these guidelines for paediatric and neonatology departments and to achieve better implementation. The secondary objective is to determine if the characteristics of professionals are related to these expectations and uses of local CPGs.

## Methods

### Study context

This study was conducted in the University hospital of Rennes and in the local hospital of Fougères. The paediatric department of the University hospital proposes two pocketbooks of local CPGs to all paediatricians (residents and attending physicians including fellows) working in the institution and in the local hospital. Each local CPG is written by a physician and then is peer-reviewed (one or several peers). Management of these local CPGs depends on one physician and is different for each sub-department (emergency, neonatology and paediatric intensive care unit). These local CPGs are available in 3 formats: on paper, on a mobile health application incorporated into smartphones and on the hospital intranet for some guidelines. Their structure is dictated by the quality department of the hospital as follows: object and purpose, responsibilities, definitions, actions and methods (diagnosis, therapy), references.

### Design

This study used a mixed-methods design with a sequential design: quantitative data collection first and then qualitative study. This sequence allows to interpret results derived from large-sample data and to better understand the findings from the first phase [9]. We followed COREQ guidelines, a 32-item checklist for explicit and comprehensive reporting of qualitative studies [10].

### Participants

There are one hundred paediatricians (residents and attending physicians) in the University hospital and the local hospital and they were all invited via e-mail to participate in the study. French medical residents complete 6 years in medical school and then choose a specialty, the residency lasts between 6 and 10 semesters depending on the specialties. Residents are certified between the middle and the end of residency. They work full time at a hospital and they have a relative autonomy: they can do prescriptions under the responsibility of an attending physician.

Paediatric surgeons, geneticists, paediatric anaesthesiologists and paediatricians who only work as consultants within our hospital system and, who do not use the local CPGs, were not included in the study.

### Quantitative study

#### Survey

Themes identified from a literature review were used to develop our 16-item survey. All participants (*n* = 100) received this survey online or in hard copy and responded anonymously. Physicians are usually in high and constant demand and to favour a high response rate, we elaborated a quick survey that can be completed in a maximum of ten minutes. Items included a 5 point-Likert scale for answering. This part of the study was conducted between January and March 2019.

#### Statistical analysis

Demographic data including sex, status, role in local CPG development, assignment, semester of training (for residents only) were summarized and compared using the Fisher exact test.

Continuous variable (age and duration of use) and coded Likert scales (− 2: strongly disagree and 2: strongly agree) were expressed as medians with interquartile ranges [25p, 75p] and compared with the Wilcoxon rank sum test. We chose an odd-numbered Likert scale of 5 points in order to offer a neutral mid-point. This avoids a forced choice and therefore a random response of one of the two points framing the omitted mid-point [11].

To identify groups of similar questions, we used a hierarchical clustering analysis on the Euclidean distance matrix formed by participant responses to the Likert-type questions. This method uses an agglomerative approach based on dissimilarity (Euclidean distance) measure between each pair of answers. We supposed an equal distance between scale points*.* We measured within-cluster similarity using the average silhouette width. Then, we selected *k* = 15 clusters of questions because of their high internal consistency based on a Cronbach’s *α* above 0.7 [12].

*P* < 0.05 was considered statistically significant for all the analysis. Data were analysed using the software R Core Team® (2018).

### Qualitative study

#### Setting and sample

Participants who had taken part in the survey were eligible to participate in the semi-structured interviews on a voluntary basis. The inclusion criteria for interview participants were based on achieving a maximum variation in our sample regarding age, professional experience and assignment. Researchers who conducted the interviews informed participants, with a letter and then face-to-face, about the aim of the study and their right to withdraw their participation at any time without giving any reason.

#### Data collection

Semi-structured interviews were conducted in French by two paediatric residents (ID and JD). Data collection spanned from May 2019 to February 2020. Each interviewer carried out repeated interviews with researchers specialised in qualitative method. These interviews were not included in the data collection. Sessions were semi-structured, with a pre-defined list of open-ended questions focused first on the current uses of the local CPGs and then on desired improvements, ease of use and open-ended suggestions. The discussion guide was developed by all authors (NG, ID, JD, NM, GC, PP and AB) after a review of the literature before starting the study. Interviews were conducted in person at a private office space located in the participants’ hospital workplace. Participants were contacted through email or face-to-face.

To ensure reliability, we used the same interview guide in every interview. Sessions were recorded with the consent of each participant and then transcribed verbatim and de-identified. The interviewers knew most of the participants prior to the study as they work in the same paediatric department but they had no hierarchical superiority with the responder and this was not a personal matter. The personal goals and reasons for the research were revealed to the participants.

All through the interview, the moderator summarised and reformulated the speech and presented them back to the participant to avoid researcher’s interpretation and respect the participants’ own points. After the session, participants completed a brief survey to gain their socio-demographic characteristics.

#### Data analysis

The analysis procedure was conducted by two persons (JD and NM) using an inductive approach to identify themes that emerged from the data. Each transcript was independently read several times to facilitate immersion in the data. Following this, the researchers used open coding process to summarise participants’ views by assigning words to quotes or paragraphs. The coding of the two researchers were then compared and in the event of any discrepancies or a disagreement, two other physicians (NG and AB) adjudicated. This method enhances the validity of the assigned categories and attempts to reduce researcher bias. A catalogue of themes and sub-themes was then created and presented in tabular form. Constant comparative analysis was used to assess overall saturation [13]. Authors collectively selected and presented verbatim quotes to illustrate the thematic findings in tabular form. We coded data from transcripts using the Saldaña method [14] and evaluated the frequency of each theme using the qualitative data management software NVivo® 12 Plus (QSR International). To ensure the reliability of the coding and analysis of the data, findings were discussed among the authors. Data were then analysed based on the participants’ status and assignment. Still using NVivo® 12 Plus (QSR International), we imported participants’ demographic characteristics and compared their responses according to these attributes.

In the last phase of the analysis, data from the quantitative survey and the interviews were interpreted together. The interviews were used to explore and complete the quantitative data or to find differences between the two datasets.

### Ethical considerations

The study was approved by the local Ethics Committee (reference number 19.147). All participants gave their informed consent before participating. A physician was responsible for explaining the research project to potential participants and to send an email newsletter about the purpose and conduct of the research.

## Results

### Phase 1: survey

A total of 83 physicians participated in the study (response rate 83%); 42.2% (*n* = 35) were residents and 57.8% (*n* = 48) were attending physicians. The median age was 36.29 years [25; 64] and 81.9% were women. Few participants were writer of local CPGs (7.23%). The median duration of use of local CPGs was 4 years [2; 11]. Characteristics of the research participants are summarised in Table 1. A Fisher test (*p* > 0.3) did not show any significant repartition between responders and non-responders for principal assignment of physicians.

**Table 1:** Characteristics of quantitative questionnaires participants

	Responders	*n*	Non responders	*n*	*p* value
Sex		83		17	
Female	68 (81.9%)		16 (94%)		0.29
Male	15 (18.1%)		1 (5.8%)		
Age (range)	36.29 [25; 64]	83	*Missing data*	17	
Status		83		17	
Resident	35 (42.2%)		3 (18%)	38	0.097
Senior	48 (57.8%)		14 (81.8%)	62	
Role in local GPGs development		83			
Only user	48 (57.8%)		*Missing data*		
Writer	6 (7.23%)		*Missing data*		
Approver	1 (1.2%)		*Missing data*		
Handler	1 (1.2%)		*Missing data*		
Writer/approver	22 (26.5%)		*Missing data*		
Writer/handler	1 (1.2%)		*Missing data*		
Principal assignment (senior)		48		14	
Paediatric haematology and oncology	5 (10%)		1 (7.1%)		0.74
Maternity	1 (2.1%)		0 (0%)		
Neonatology	5 (10%)		0 (0%)		
General paediatric	18 (37%)		8 (57%)		
NICU*	5 (10%)		1 (7.1%)		
PICU*	3 (6.2%)		2 (14%)		
Paediatric emergency	11 (23%)		2 (14%)		
Semester (Residents)	5.00 [2.00; 11.00]	35	7.00 [5.00; 8.00]	3	
Duration of use of local GPGs (years)	4.00 [2.00; 11.00]	83	*Missing data*		

*NICU Neonatal intensive care unit, *PICU* Paediatric intensive care unit

The majority of participants (97%) reported using local CPGs sometimes on hard copy, 42% sometimes on intranet and 48% sometimes on smartphones. The existing local CPGs were considered diagnostic-oriented for 34% of the physicians and therapeutic-oriented for 82% of the physicians. About a quarter of the participants (28%) expressed the wish for more local CPGs for diagnosis purposes, and almost half (53%) expressed this wish for therapeutic purposes. Almost all participants (98%) wanted references based on international guidelines and 69% explicit references in local CPGs. Answers were not polarised for the question “a good guideline should be based on local guidelines or usual practice in unit”. Few participants (18%) felt that a good guideline should be based on physician experience. This cluster of questions showed a high internal consistency (*α* = 0.8 [0.72; 0.88]). The main objective of a local CPG should be therapeutic according to 80% of the participants and 70% used local CPGs to write prescription. Only 12% of the participants used local CPGs to make a diagnosis. About three-quarters of respondents (77%) reported using only some parts of the guideline they found interesting and not the entire guideline. For the question “I do not (always) implement the local CPG” answers were not polarised. However, there was a high internal consistency (*α* = 0.73, 95% CI [0.62–0.85]).

Physicians’ awareness of the grade of evidence was found to be mixed and answers were not polarised for this question. Almost half of the respondents (47%) considered grade of evidence when using and/or reading local CPGs. There was a high internal consistency in answers for these two questions (*α* = 0.78–95% CI [0.69–0.87]).

The use of local CPGs was mainly reported in those working in the emergency ward (60%). A high percentage of participants were interested in receiving notification when local CPGs were updated (82%) and were in favour of having local CPGs reviewed and approved by both a resident and an attending physician (61.5%).

Physicians mostly (78%) read just the part of the local CPG they were interested in and only 28% read the whole local CPG (*α* = 0.5–95 CI [0.28–0.71]).

Concerning the clarity of information contained in local CPGs, almost all the responders (97%–95 CI [90–99%]) considered decision trees as the best method for relaying the information and 81% thought they were better than text (*α* = 0.48–95 CI [0.28–0.69]). With regard to the classification of local CPGs, 23% of participants would like to see them classified by degree of severity and urgency and 50% did not express an opinion on this point. Finally, more than half of physicians (69%) had already searched for a protocol that did not exist.

Hierarchical cluster analysis showed that 8 out of 15 clusters had a Cronbach’s alpha above 0,5 and that 4 clusters had this value above 0,7. Internal consistencies were stronger in the following clusters: local CPGs supported by local guidelines, medical unit habits and physician experience.

### Phase 2: qualitative study

Twenty-four physicians participated in the interviews. Almost all of them (*n* = 22) were women, 19 used local CPGs very often and 5 used it maximum once a week or less. “Very often” was defined by responders themselves as "daily" or "several times a week". Table 2 shows the characteristics of participants. Each interview lasted between 12 and 24 min. Data saturation was obtained because we did not find a new theme after several analyses of the data. Analysis revealed five emergent themes: improvement of local CPGs, patterns of use, reasons for non-implementation, alternative sources and perspectives (Table 3). In the second part, we analysed qualitative data based on participants’ status and assignment.

**Table 2:** Characteristics of semi-structured interviews participants (*n* = 24)

		*n*
Sex		24
Female	22 (91.6%)	
Male	2	
Age (range)	33.3 [26; 53]	
Status		24
Resident	10	
Senior	14	
Role in local GPGs		24
User	24	
Writer	10	
Approver	7	
Handler	1	
Writer/approver	7	
Writer/handler	1	
Principal assignment (senior)		14
Paediatric haematology and oncology	2	
Maternity	1	
Neonatology	2	
General paediatric	2	
NICU*	3	
PICU*	0	
Paediatric emergency	4	
Semester (residents)	5.3 [3.00; 8.00]	10
Duration of use of local GPGs (years)	6.58 [1.00; 24.00]	24

**Table 3:** Emerging themes with subthemes and exemplar quotations

Themes	Subthemes	Representative quotations
Improvement of local protocols of care	Missing GPGs or part of GPG concerning the following and the monitoring once the patient is admitted in a care unit, after the emergency	Hum, yes yes, for sure during night shift…hum, we use it frequently, otherwise, hum, every day. (Physician no 1)
	Adaptation to the local structure	Not necessarily easy to apply, for example when the drugs listed for the disease are not available at the pharmacy, sometimes there are some inconsistencies between what is recommended and what is available. (Resident no 6)
	To update local GPGs and notifications each time there is an update or the creation of a new local GPG	And to have an alert when a new protocol is introduced in the app, with…hum…I don’t know, a little message “protocol updated”, hum, with the possibility to consult it. (Resident no 5)
	Better ranking of the GPGs	Well, paper, maybe it would be, well, classify protocols, I mean to make more sense, listing in order of topic or even alphabetically. Maybe more by topics, because now I feel like everything is mixed. (Senior no 4)
	To extend the local GPGs writers to residents to have another point of view	Maybe concerning the development, it can be good to discuss it with residents. Well, because we use it also, we could…have a say, well if it seems clear for us, the way it’s conceived. Same thing for the use. (Resident no 6)
Patterns of use	Mainly when physicians were going on night shift and also during daily practice	Hum, yes yes, for sure during night shift…hum, we use it frequently, otherwise, hum, every day. (Physician no 1)
	Verification for prescription, especially when it concerns an uncommon disease and also to check the general attitude	But I check every time the treatment, even if after a while I get to know it, I check if the posology is right and it is the same for the additional exams, make sure I remember everything. (Resident no 1)
	Education use of protocols with juniors	For most part of very standardized protocols like diabetes, I always look at it to make sure I don’t miss anything. (Physician no 6)
		On a less frequent or usual reason for consultation, something I have no expertise on. (Physician no 13)
		Well, even if I know it, every time I go back to watch and show the residents…which protocol. We use it in a pedagogical way also.(Physician no 1)
Reason for non-implementation	The clinical context was a barrier if it was not a typical situation matching the protocol	I don’t know, for example social context, the treatment implementation, of course it’s a little bit of a mismatch between recommendation and what we’re going to choose for the child. It can be an antibiotic that we could prescribe orally but we believe it will not be given. In that case, we can keep the child to treat him parenteral or this kind of things. (Resident 10)
	The lack of updating generated a mistrust of the protocols and physicians felt they lost time checking the existence of new guidelines	I think it is also due to old protocols, thus I pay more attention and I don’t follow the whole protocol and so it seems incomplete.(Physician no 4)
	Some protocols are not easy to understand and sometimes residents feel they have contradictory advices from seniors	For example, the guideline concerning metabolic diseases, it is very…well it’s kind of difficult to apprehend it, when we look for an information I think, it’s a little long. It’s not that it is disorganised, but I have the feeling that it is difficult to find information in it. Well, there are also some protocols, for example, well I think of those concerning the hydroelectrolytic inputs, well some protocols like this. It is very very detailed, a little too much, concerning physiopathology or concerning the…the…the articles and bibliography on which they are based and the major information are not apparent. (Resident no 6)
		Well sometimes in some units, I observed they did things differently from the protocol, sometimes I saw some chiefs criticizing the protocol, so…hum, I have learned that we had to do things differently. (Resident no 3)
Alternative sources		Sometimes when there is nothing in the local GPGs, I am going to check on the internet or on the Scientific Societies. (Physician no 8)
Perspectives	Frequent updating of protocols and removal of outdated protocols	Then, hum…that they be updated because some local guidelines are very old and no longer relevant so, something…updated with the latest current recommendation (Physician no 6)
	Senior physicians wondered if it was adapted to extend protocols beyond the University hospital of Rennes and if this option was selected what the responsibility would become	We are sometimes called by peripheral hospital, local GPGs are about local habits from Rennes and I’m uncomfortable with the availability for other hospitals. (Physician no 7)
	The idea of a national protocol to standardize practices on a national scale	So I don’t know if it is to be broadcast, already broadcast it in the university hospital, I find it great. Maybe when there are some regional referrals for example, for some sub-specialties, maybe it’s worth it to broadcast it. It was a point for reflection. (Physician no 4)
	Combination of prescription software with protocols	Updating or national pocket books. The attitude should not be different in Marseille, Rennes or Paris. (Senior no 9)
	Missing protocols concerning paediatric sur-specialties	It would be great to combine local GPGs with a prescription software. (Physician no 7)
	Clear summary and and logical way of categorization	Now there are some missing sub-specialties, common things, we know that we’d use it every day and it is not there. (Resident no 9)
		It has to be first handling of a cardiac arrest first, then pain, etc. Just an idea. (Physician no 9)

#### Participants’ status and assignment

**Fig. 1:**
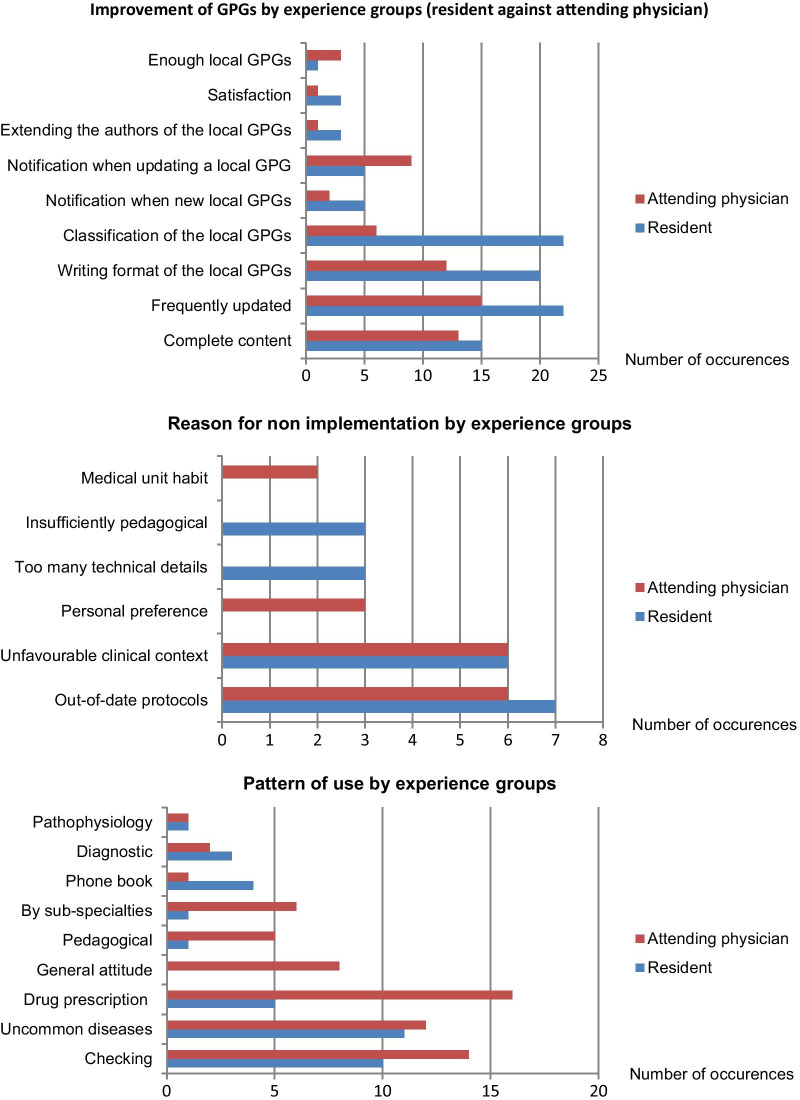
Verbatim analyse depending on the status and assignment

We analysed associations between attitudes, such as reason for non-implementation, improvement and pattern of use, toward local CPGs and physicians’ characteristics. No major differences by status or assignment were found in this analyse. In terms of patterns of use, the local CPGs were mainly used for verification and prescription regardless of status or assignment (Fig. 1).

## Discussion

CPGs are designed to improve standardization and quality of care. CPGs allow evidence-based practice to integrate faster routine care [15]. In this study, we explored the expectations of physicians regarding paediatrics local CPGs. Quantitative and qualitative approaches showed that the characteristics of professionals were not related to the expectations and uses of local CPGs. Francke et al. published a meta-analysis to gain a better understanding of the factors affecting guidelines implementation. Age and/or experience were found to be determinants of guidelines use with younger or less experienced professionals being more likely to use guidelines than older and experienced professionals [1]. Our results are not in line with these findings as in our sample, CPGs were widely used by both residents and attending physicians. However, in the qualitative part of the study attending physicians also reported that they sometimes relied on their department common practices.

Participants also expressed a desire to have local CPGs based on international guidelines with high grade of evidence. This is reflected in literature that adherence to evidence-based guidelines appears to be higher for guidelines with a clear scientific basis [16]. In another study, it is mentioned that quality of practice guidelines is directly related to the quality of medical evidence supporting the recommendation [17]. The GRADE system details in four points how to determine the strength of a recommendation [18].

In our study, participants were mainly interested in treatment CPGs. Similar findings were found in a German study which explored perception of guidelines (therapeutic and diagnostic) in a group of primary care physicians. Therapy management was reported as one of the most important aims for this group [19].

Even if participants declared to be mainly satisfied with the available CPGs, improvement of the local CPGs was the major theme identified. The physicians interviewed indicated that local CPGs needed to be better adapted to the local structure and updated as often as possible. However, among physicians, the reported frequency at which local CPGs should be updated was very different. A study provided an empirical estimate of the rate at which guidelines become outdated and it was found that guidelines should be reassessed every 3 years [20]. A cohort of 134 published (National Institute for Health and Care Excellence (NICE) clinical guidelines was analysed and found that 86% of guidelines were still up to date 3 years after their publication and that the median life span from its publication to the date when a decision was made to update was 5 years [21]. The importance of keeping guidelines up-to-date and updating parts of it rather than re-writing the whole guideline was underlined in another study. An ongoing monitoring system should be created by guideline developers to implement a systematic updating procedure depending on the guideline’s topic and the organizational capacity to enable rapid adaptations to current events [22].

The present study also identified the interest in extending local CPG writers to trainees and especially residents to introduce a different perspective. Guidelines developed by target groups and experts have been previously found to increase the chances of successful implementation [1]. Another study suggested that guideline development groups should be well balanced in terms of the selected disciplines to avoid dominance of those who are likely to be perceived to have higher power status [23].

Physicians were found to primarily use local CPGs for verification when prescribing, especially for uncommon diseases. Previous research that has studied the use of guidelines and protocols in a different setting from our study (hospice care) found that their use was mainly for pharmacological treatment [24]. Audi et al., updated a list of the 100 of the most commonly prescribed drugs in England and emphasized the importance of using standardised prescription forms as a tool to improve prescribing skills [25].

The identified barriers to the implementation of local guidelines were the clinical context including situations that differ from the guideline and, again, the lack of updating. Bergman et al. [17] note that guidelines in general are not modified to meet patient and site-specific needs. By collecting local outcome data, physicians are able to customise guidelines through the collection and feedback of this data from their population.

Residents raised the issue of difficulty in understanding CPGs and of sometimes conflicting recommendations from their attending physician. A study mentioned that rewriting guidelines to increase behavioural specificity may be the simplest and most effective method to increase implementation, by providing clearer information about requirements and improving the sense of doing well [26]. Cabana et al. detailed barriers to CPGs adherence including: lack of awareness and familiarity with CPGs; low outcome expectation from CPGs; accessibility; clinical time pressure and limited resources to implement recommendations [15]. Guideline adherence seemed better when reviewed with local leaders. A study proposed a stepwise procedure to increase physicians’ compliance with guidelines by adapting the recommendations to the target setting and context of use, taking into account the organization of the health care system and cultural context [7].

Physicians interviewed in our study used local CPGs also as a pedagogical tool for teaching residents. This finding is in accordance with a study that evaluated the interest of a formulary in drug prescribing and particularly in a medical student population [27]. The results of this study showed that using a formulary increases the competence of medical students in rational prescribing.

Physicians would like to see outdated CPGs more frequently updated or removed. This is confirmed by the Appraisal of Guidelines for Research and Evaluation (AGREE II) collaboration that a procedure for updating guidelines should be provided for a better implementation [28]. This rigor is an ongoing challenge. Actually, lack of time was a barrier often expressed by the local CPGs writers interviewed in our study and it was a reason for delaying updates.

Ciquier et al., emphasized that when implementing CPGs, it should be anticipated that CPGs will need to be appraised, monitored and possibly revised, in order to ensure applicability [29].

Use of electronic format may overcome some of the implementation barriers noted with the use of notifications as well as patient-specific recommendation based upon the integrated data at the time of the patient visit. Physician use and compliance with CPGs is improved when a computerized format is provided [30]. When information is accessible with minimal need for searching, information integration is likely to proceed in a perception-like, holistic manner [31].

## Limitations and strengths

Our study was limited in several areas. Some answers were polarised (extreme responses on Likert scales) and some questions had a low internal consistency making it impossible to explore the corresponding answers. We selected 15 clusters of questions using the hierarchical clustering analysis and 5 clusters contained only one question. We could not analyse internal consistency for these 5 clusters and maybe they have a validity anyway. We did not explore the semantic of the local CPGs as our interview guide did not focus on this aspect of the CPGs. The French National Authority for Health has established that clinical practice guidelines should preferably be in electronic formats and take into account modern technological tools. Access should be straight forward lists with bullet points and decision trees should be provided as well as links to access reports and other documents. Another limitation of our study was the study sample comprised only of physicians (paediatricians only). It is not a multidisciplinary study because it does not involve all potential users of the guidelines. However, literature showed that specialty does not appear to be a significant factor in guideline adherence and suggested it is not necessary to target specific groups during development of practice guidelines [32].

Despite these limitations, recurring themes were clearly established, and our population included a wide diversity of profiles. The response rate to the quantitative survey was high, about 80%. Moreover, high internal consistency validates the good quality of the quantitative survey and answers obtained. The majority of users declared using local CPGs very often so there is an interesting level of critical thinking from the panel.

The mixed approach found consistent results and this probably empowers the level of evidence of the study. Quantitative approach highlighted several inadequacies in local CPGs as evidenced by the high number of polarised answers. Qualitative analysis enabled us to gain a deeper understanding and to clarify the answers that were not polarised (response neutral on Likert scale).

The generalizability of our findings is limited by the response rate and the sampling from only two hospitals of the region. Because recruitment was on a voluntary basis it is possible that those who completed the survey had stronger feelings about the topic however it was noted that respondents had similar characteristics to non-respondents.

## Conclusion

We explored physicians’ expectations concerning the paediatrics local CPGs.

Our findings highlight that there is still a lot to be done in improving implementation of guidelines, therefore, promoting better outcomes for patients. Using the above-mentioned literature, we can suggest some interesting approaches to consider when developing local CPGs and for increasing their implementation (See Table 4). It is necessary to focus on therapeutic information and to classify items by specialties in order to have a quick access to the searched guideline. When it comes to the redaction of the local CPGs, it is important to justify local CPGs with international guidelines, while adapting them to the local healthcare setting, as well as listing the sources used and promoting double validation of local CPGs with a trainee and an attending physician.

**Table 4:** Quality criteria for local good practice guidelines

Focus on therapeutic information
Classify items by specialties in order to have a quick access to the searched guideline
Justify local GPGs with international guidelines and adapt them to the local healthcare setting
Mention the sources, ideally with multiples sources, to reduce necessity to look for other references
Promote double validation of GPGs with a resident and an attending physicians
Keep local guidelines updated with an evaluation every 3 years

Frequency of updates and classification of guidelines, enabling for an easy and quick search, remain a major challenge in improving guideline adhesion. Local CPGs developers should define a systematic updating procedure before the guideline dissemination. Electronic format and mobile application could be tools to improve these shortcomings as it would enable the use of automated notifications or a keyword search system to provide rapid access to information. Another suggestion would be the existence of a crosstalk between local guidelines and prescribing software with the possibility to alert an outdated prescription.

## Supplementary Information

**Additional file 1.** Hierarchical clustering.

**Additional file 2.** Questionnaires.

**Additional file 3.** Consolidated database.

## Data Availability

The data supporting the findings are contained within the manuscript and its Additional files 1, 2, 3: Supplementary. All verbatims of the semi-structured interviews analysed during the current study are available from the corresponding author on reasonable request.

## Abbreviations

ISO: International Organization for Standardization
CPGs: Clinical practice guidelines
COREQ: Consolidated criteria for reporting qualitative research
