# Correction to: Criteria for assessing the quality of clinical practice guidelines in paediatrics and neonatology: a mixed-method study

**DOI:** 10.1186/s12911-021-01641-4

**Published:** 2021-10-05

**Authors:** Joanna Dagard, Nadia Mazille-Orfanos, Nawras Georgi, Intissar Dechicha, Guy Carrault, Patrick Pladys, Alain Beuchée

**Affiliations:** 1 Department of Pediatrics/Neonatology, CHU Rennes, 35033 Rennes, France; 2 LTSI-UMR_S 1099, Univ Rennes, Inserm, 35000 Rennes, France; 3 Paediatric Department, Hospital of Fougères, Fougères, France

## Correction to: BMC Med Inform Decis Mak (2021) 21:269 https://doi.org/10.1186/s12911-021-01628-1

Following publication of the original article [1], it was reported that the names of all authors were published with their given name and family name in reverse order. The correct authorship list is given in this Correction and the original article [1] has been updated.
