# Supplementary figures and images for: Criteria for assessing the quality of clinical practice guidelines in paediatrics and neonatology: a mixed-method study
Source: BMC Med Inform Decis Mak. 2021 Sep 21;21:269. doi: 10.1186/s12911-021-01628-1 (PMC8456649; doi:10.1186/s12911-021-01628-1)

Cluster Dendrogram  
Hierarchical clustering (euclidian distance)

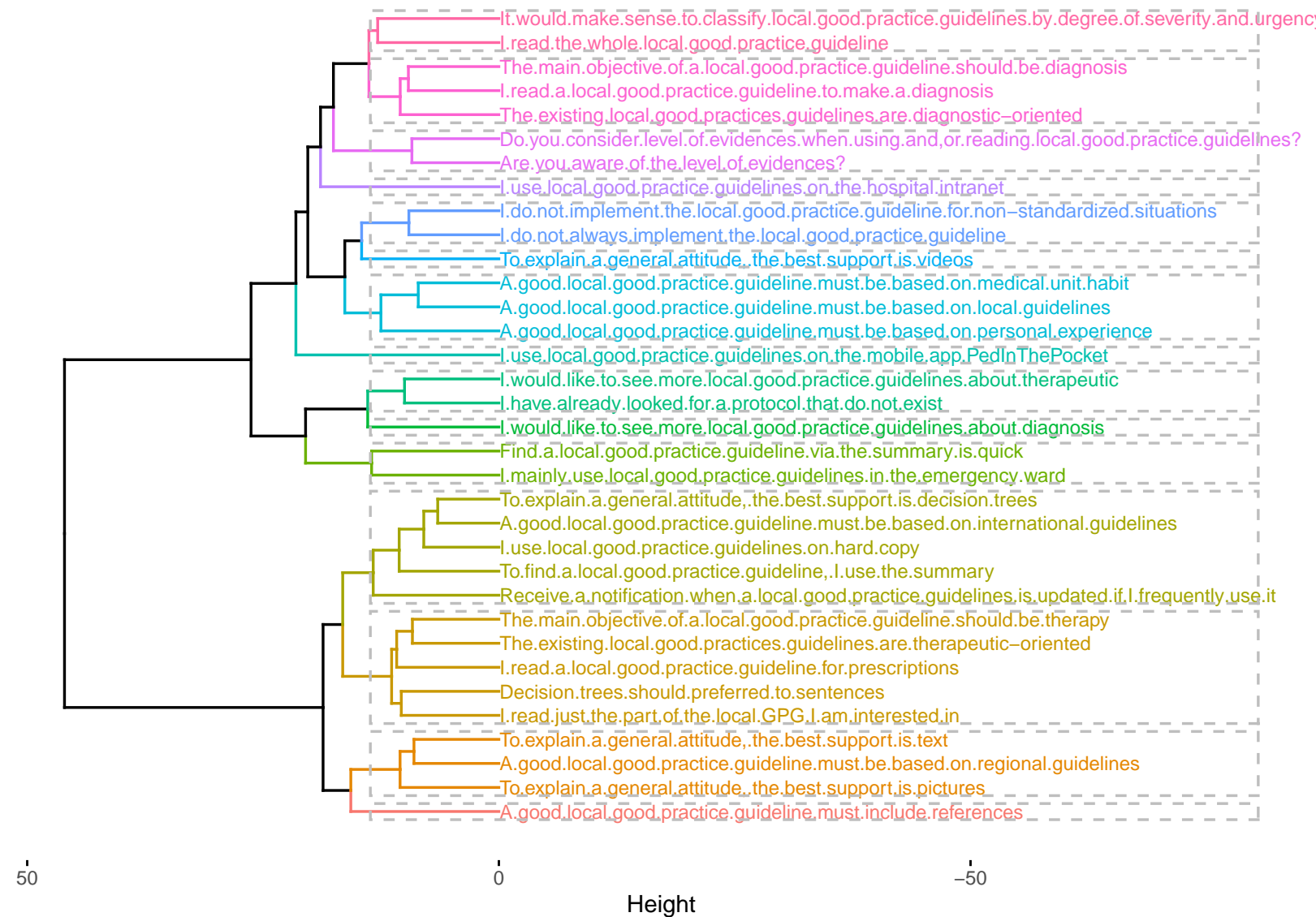

Supplement: Supplementary file 2 — Additional file 2. Questionnaires. [file 12911_2021_1628_MOESM2_ESM.pdf]
