# Supplementary material for: Criteria for assessing the quality of clinical practice guidelines in paediatrics and neonatology: a mixed-method study
Source: BMC Med Inform Decis Mak. 2021 Sep 21;21:269. doi: 10.1186/s12911-021-01628-1 (PMC8456649; doi:10.1186/s12911-021-01628-1)
Supplement: Supplementary file 3 — Additional file 3. Consolidated database. [file 12911_2021_1628_MOESM3_ESM.docx]

**Characteristics of participants:**

Mail address

Gender

Birth year

Status

Role in local good practice guidelines

Principal assignment

Semester for resident

Are you currently using local good practice guidelines? If yes, for how long?

**Quantitative questionnaire:**

I use local good practice guidelines on hard copy

I use local good practice guidelines on the hospital intranet

I use local good practice guidelines on the mobile app PedInThePocket

I mainly use local good practice guidelines in the emergency ward

The existing local good practices guidelines are diagnostic-oriented

The existing local good practices guidelines are therapeutic-oriented

To find a local good practice guideline, I use the summary

Find a local good practice guideline via the summary is quick

I read a local good practice guideline for prescriptions

I read a local good practice guideline to make a diagnosis

I read the whole local good practice guideline

I read just the part of the local GPG I am interested in

I have already looked for a protocol that do not exist

I would like to see more local good practice guidelines about diagnosis

I would like to see more local good practice guidelines about therapeutic

I do not always implement the local good practice guideline

I do not implement the local good practice guideline for non-standardized situations

Are you aware of the level of evidences?

Do you consider level of evidences when using and/or reading local good practice guidelines?

Decision trees should preferred to sentences

The main objective of a local good practice guideline should be diagnosis

The main objective of a local good practice guideline should be therapy

A good local good practice guideline must include references

A good local good practice guideline must be based on international guidelines

A good local good practice guideline must be based on regional guidelines

A good local good practice guideline must be based on local guidelines

A good local good practice guideline must be based on medical unit habit

A good local good practice guideline must be based on personal experience

To explain a general attitude, the best support is text

To explain a general attitude, the best support is pictures

To explain a general attitude, the best support is decision trees

To explain a general attitude, the best support is videos

It would make sense to classify local good practice guidelines by degree of severity and urgency

Receive a notification when a local good practice guidelines is updated if I frequently use it

A local good practice guideline should be approved by an expert

A local good practice guideline should be approved by a resident

A local good practice guideline should be approved by an expert and a resident

**Qualitative questionnaire:**

When did you start to use local good practice guidelines?

In which situations are you using them?

Could you explain how you are using them? Which steps do you follow?

How often do you use them: occasionally, very often?

Do you use the whole local good practice guidelines or just a precise part?

What are you looking for in the local good practice guidelines? Is it depending on situations?

Do you implement the whole local good practice guidelines or just a precise part?

If you do not implement a local good practice guideline, why is it for?

Are there some situations where the local good practice guidelines are more useful to you?

Is the book of local good practice guidelines complete today?

Does it meet your expectations?

Do you find procedures complete?

Do you need to complete local good practice guidelines with other sources?

Do you find local good practice guidelines easy to apply?

According to you, which format should be used for local good practice guidelines?

Do you have any difficulty using the local good practice guidelines?

Which improvements would you like for the local good practice guidelines?

Were you bothered with the frequency of update of the mobile app? If yes, how often would you like an update?
